# Supplementary material for: Rethinking a Non-Predominant Pattern in Invasive Lung Adenocarcinoma: Prognostic Dissection Focusing on a High-Grade Pattern
Source: Cancers (Basel). 2021 Jun 4;13(11):2785. doi: 10.3390/cancers13112785 (PMC8200026; doi:10.3390/cancers13112785)
Supplement: Supplementary file 1 [file cancers-13-02785-s001.zip › cancers-1235919-supplementary.pdf]

cancers

MDPI

Article

Rethinking a Non-Predominant Pattern in Invasive Lung Adenocarcinoma: Prognostic Dissection Focusing on a High-Grade Pattern

Yeonu Choi, Jonghoon Kim, Hyunjin Park, Hong Kwan Kim, Jhingook Kim, Ji Yun Jeong, Joong Hyun Ahn and Ho Yun Lee

Supplementary Data 1. Imaging acquisition parameters

DECT parameters were as follows: 105 mAs at 140 kV, 248 mAs at 80 kV; beam pitch, 0.7; gantry speed, 0.5 sec per rotation; pixel matrix, 512 × 512; and collimation, 32 x 0.6-mm. From DECT imaging, three datasets were produced: 80 kV, 140 kV, and enhanced weighted-average images that were produced by combining the 80-kV and 140-kV datasets with a weighting factor of 0.6 (almost 120 KV).

Table S1. Clinical and pathologic characteristics.

Characteristic	No. of patients (n = 275) or No. of tumors (n = 290)	%
Age (mean ± SD) (years)	60 ± 9	
	Sex	
Male	129	46.9
Female	146	53.1
	Smoking	
Non-smoker	187	68.0
Ex-smoker	68	24.7
Current smoker	20	7.3
	T status	
pT1	215	78.2
pT2	54	19.6
pT3	6	2.2
	N status	
pN0	247	89.8
pN1	10	3.6
pN2	18	6.6
	Operation type	
Wedge resection	50	17.2
Segmentectomy	46	15.9
Lobectomy	194	66.9

Note. SD = standard deviation.

Table S2. Univariate analysis for factors affecting disease-free survival.

Variable	Univariate		
	OR	95% CI	P value
		Shape	
Diffuse	0.505	0.287-0.890	0.018
Taller than wide		Ref	
		Area 30	
Compact		Ref	
Diffuse	0.330	0.187-0.582	0.001
		VNC group	

Upper		Ref	
Middle	0.282	0.135-0.587	0.001
Lower	0.145	0.051-0.407	0.000
	Second most predominant pattern		
Low		Ref	
Intermediate	1.598	0.791-3.230	0.192
High	4.276	1.966-9.300	0.000
	GGO percentage		
<25%		Ref	
25%≤, <50%	0.295	0.091-0.961	0.043
50%≤, <100%	0.173	0.061-0.486	0.001
100%	0.305	0.128-0.727	0.007
SUVmax	1.110	1.052-1.172	0.000
	Pathologic stage		
Ia		Ref	
Ib	3.313	1.526-7.192	0.002
IIa	7.549	2.964-19.223	0.000
IIb	17.884	6.505-49.164	0.000
III	10.498	4.631-23.799	0.000

Note. ⁻CI = confidence interval, Ref = reference, VNC = virtual non-contrast.

Table S3. Disease-free survival according to most predominant pattern.

Most Predominant Pattern	No. at Risk	Event	3 Year DFS (95% CI)	5 Year DFS (95% CI)
Lepidic	48	3	0.979 (0.938–1.000)	0.957 (0.900–1.000)
Acinar	183	35	0.889 (0.844–0.936)	0.834 (0.781–0.892)
Papillary	31	4	0.931 (0.843–1.000)	0.887 (0.772–1.000)
Micropapillary	8	1	0.875 (0.673–1.000)	N/A
Solid	20	5	0.895 (0.767–1.000)	0.739 (0.540–1.000)

Note. The log rank test could not reject the null hypothesis of the difference of DFS between five most predominant histologic patterns. CI = confidence interval, DFS = disease-free survival, N/A = not available.

Table S4. Distribution of pathologic N-positive tumors according to second most predominant pattern.

Second most predominant pattern	cN0->pN1	cN0->pN2	cN1->pN2	cN2->pN2	Upstaging (Total N-positive) No.
Low	3			2	3 (5)
Intermediate		2	3	1	5 (6)
High	4	1	1	3	6 (9)

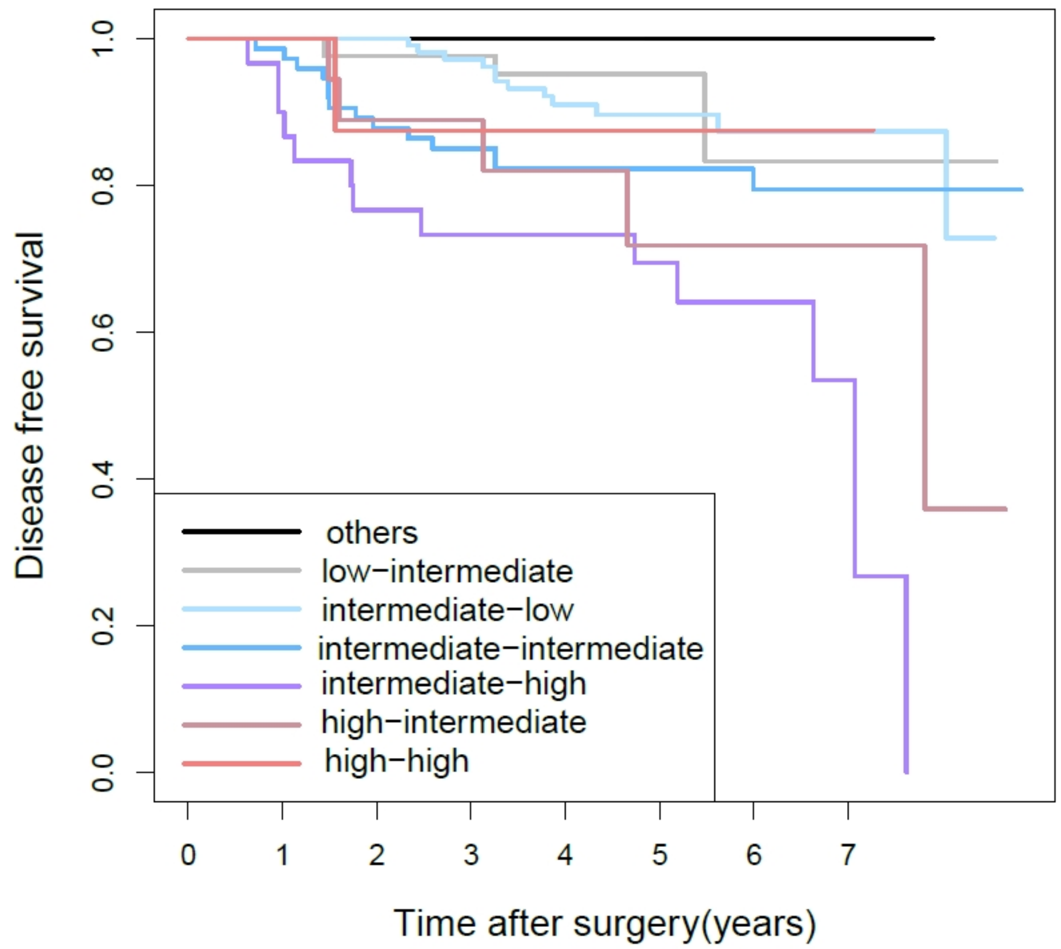

Figure S1. Disease-free survival curves for eight histologic combination groups of lung adenocarcinoma. Patients were stratified according to histologic subgroup (most predominant and second most predominant subgroup). Survival curves were significantly different among the eight groups (p = 0.04). The others group includes histologic combination of low-low and high-low grades of prognostic significance.

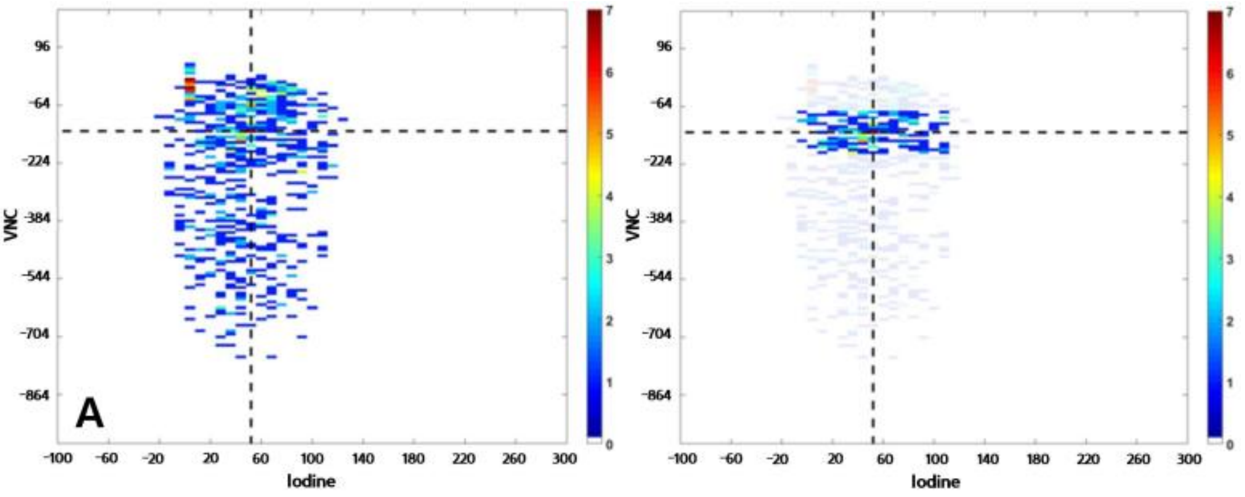

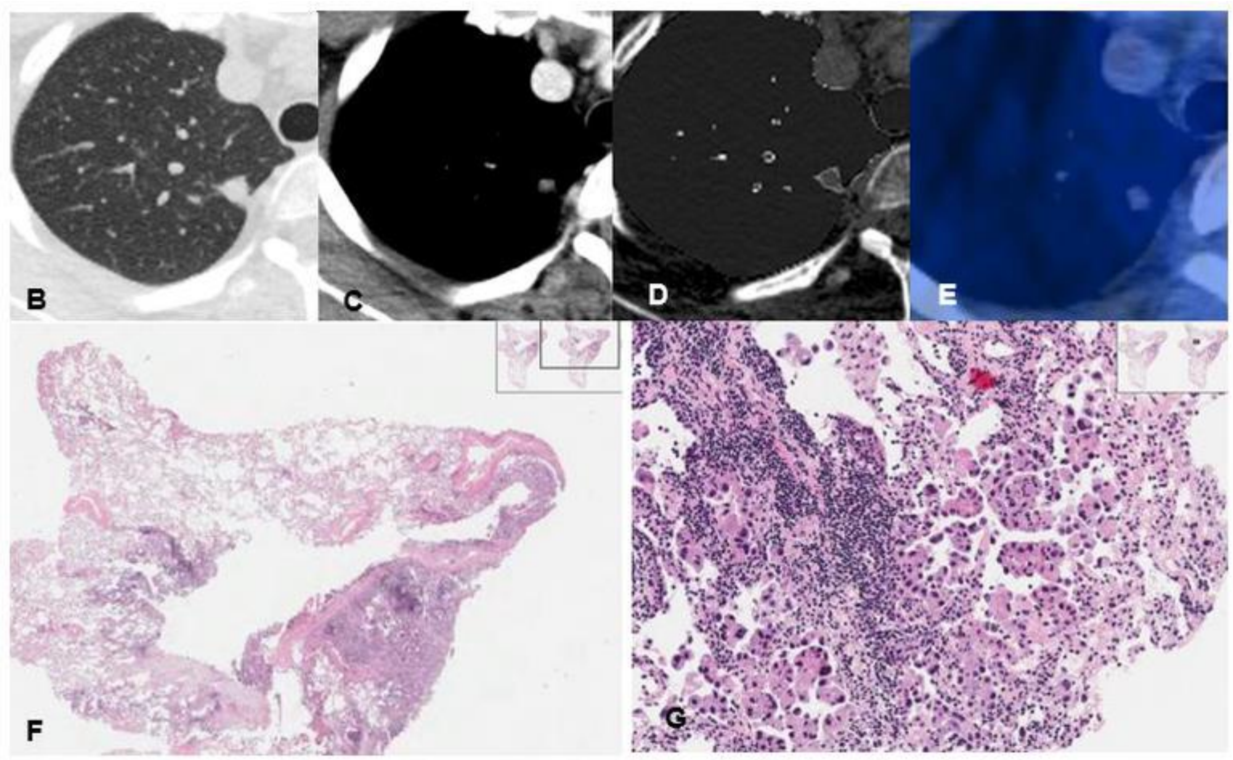

Figure S2. A 50-year-old woman with lung adenocarcinoma with a 75% papillary and 25% micropapillary pattern. (**A**) Joint histogram that shows a taller than wide shape and scattered distribution pattern. Targeted view of lung window VNC image (**B**) and mediastinal window conventional enhanced image (**C**) show a 9-mm-sized solid nodule in the right upper lobe. (**D**) On an iodine map, the range of mean iodine values was −21 to 121. (**E**) PET/CT image shows minimal FDG uptake with an SUVmax of 0.5. (**F**) Photomicrograph (HE, 10×) shows invasive adenocarcinoma with a papillary predominant pattern. (**G**) High magnification (HE, 100×) of a minor portion of the specimen shows micropapillary pattern.

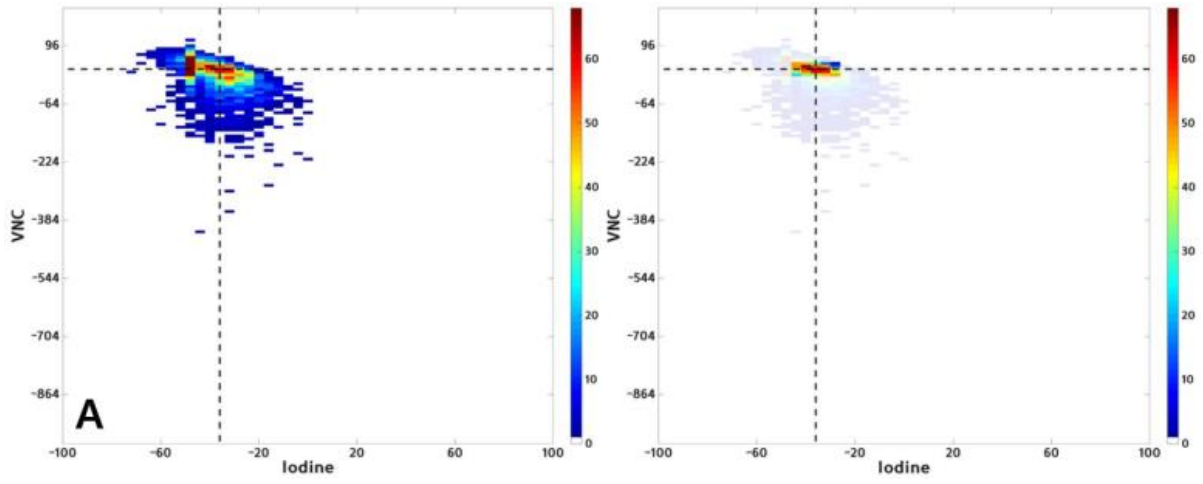

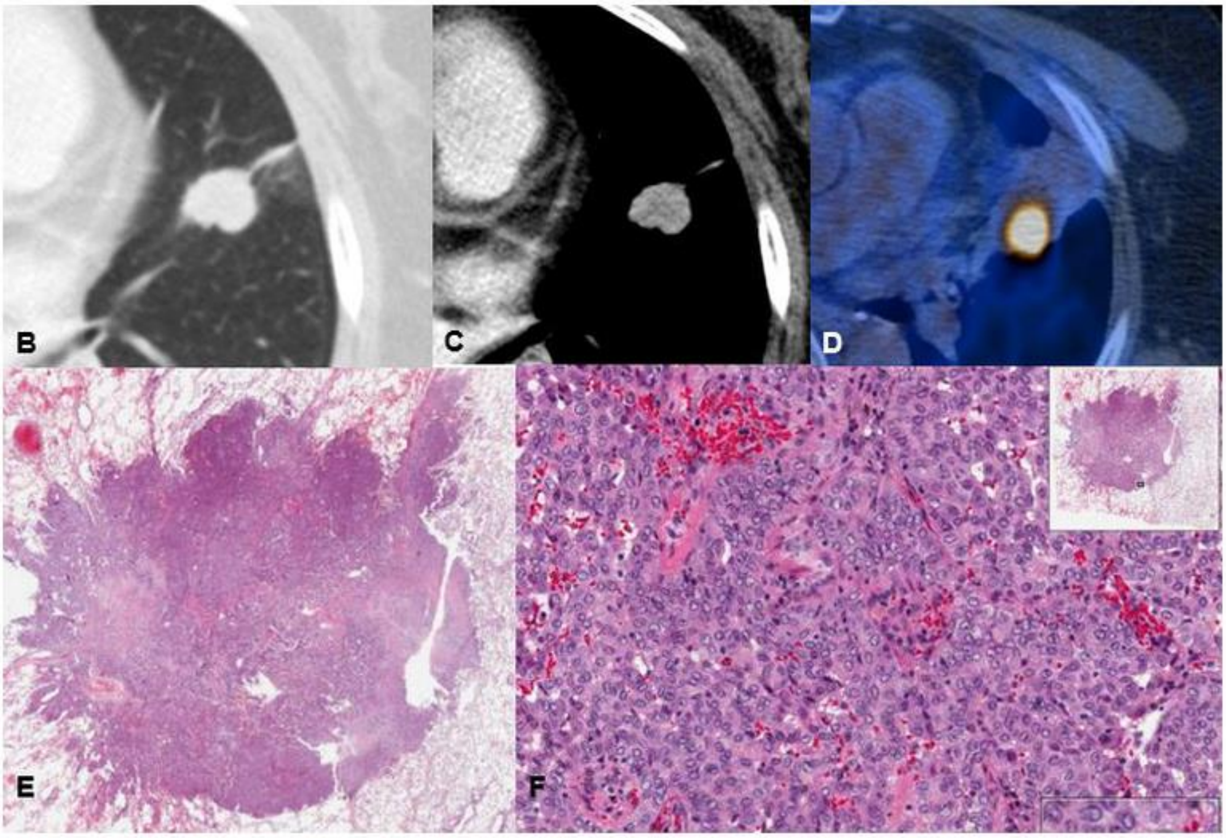

Figure S3. A 59-year-old woman with lung adenocarcinoma with a 50% acinar and 45% solid pattern. (**A**) Joint histogram that shows a diffuse shape and compact distribution pattern. Targeted view of lung window VNC image (**B**) and mediastinal window conventional enhanced image (**C**) show a 20-mm-sized solid nodule in the left upper lobe. (**D**) PET/CT image shows intense FDG uptake with an SUVmax of 10.6. (**E**) Photomicrograph (HE, 10×) shows invasive adenocarcinoma with similar portions of acinar and solid patterns. (**F**) High magnification (HE, 100×) of the specimen shows a solid pattern.

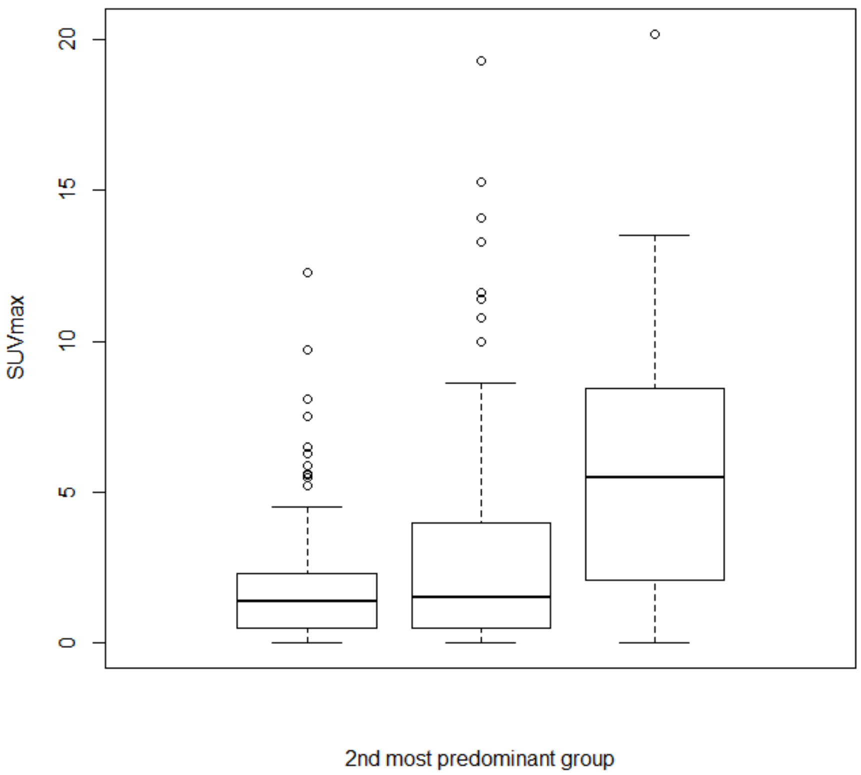

Figure S4. Distribution of SUVmax for second most predominant group when the most predominant pattern is intermediate grade. The high-grade second most predominant group showed significantly higher SUVmax than the low- and intermediate-grade groups ($p < 0.0001$).
